# Disparities in hysterectomy-corrected endometrial cancer incidence trends by histologic subtype among racial/ethnic groups in California, 2012–2019

**DOI:** 10.1016/j.ygyno.2025.04.581

**Published:** 2025-04-22

**Authors:** Jingjing Xie, Frances B. Maguire, Brenda M. Hofer, Julianne J.P. Cooley, Hui A. Chen, Arti Parikh-Patel, Theresa H.M. Keegan

**Affiliations:** a Graduate Group in Epidemiology, University of California Davis, Davis, CA, United States of America; b California Cancer Reporting and Epidemiologic Surveillance Program, University of California Davis Comprehensive Cancer Center, Sacramento, CA, United States of America; c Department of Gynecology Oncology, University of California Davis Comprehensive Cancer Center, Sacramento, CA, United States of America; d Center for Oncology Hematology Outcomes Research and Training (COHORT), University of California Davis Comprehensive Cancer Center, Sacramento, CA, United States of America

**Keywords:** Racial/ethnic disparities, Incidence trends, Endometrial cancer, Endometrioid, Non-endometrioid, Histologic subtype

## Abstract

**Background.:**

Hysterectomy-corrected endometrial cancer incidence among racial/ethnic minority groups by histologic subtype and age group has not been well studied. To examine recent trends in hysterectomy-corrected endometrial cancer rates among California women by histologic subtype, race/ethnicity, and age group.

**Methods.:**

We estimated hysterectomy prevalence from the Behavioral Risk Factor Surveillance System. Hysterectomy-corrected age-standardized endometrial cancer incidence rates (per 100,000 women) by endometrioid and non-endometrioid subtypes, age at diagnosis, and race and ethnicity from 2012 to 2019 were calculated using California Cancer Registry data. Incidence rates and annual percentage changes (APC) were estimated.

**Results.:**

Among endometrioid subtypes, American Indian women had the highest incidence (62.9 per 100,000). Incidence rates also significantly increased among Asians/Pacific Islanders (1.69 %), with an increase of 7.14 % and 7.39 % for women aged 45–54 and 55–64, respectively, though these did not reach statistical significance. In addition, Hispanics had an increased incidence rate (3.02 %) from 2012 to 2019, with a particularly sharp rise (18.42 %) observed in Hispanics aged 25–34 years between 2016 and 2019. For non-endometrioid subtypes, non-Hispanic Blacks had the highest incidence (29.4 per 100,000), with the ≥65 age group showing an upward trend (9.39 % increase from 2012 to 2016) before significantly declining by 8.16 % from 2017 to 2019. American Indians had the second-highest incidence (14.1 per 100,000), but no significant trend was observed, likely due to the small sample size of this population.

**Conclusions.:**

Our findings show that race/ethnicity is associated with endometrial cancer incidence and underscore the importance of jointly examining racial/ethnic disparities with age and histologic subtype.

## 1. Background

Endometrial cancer, the most common gynecologic cancer [1], includes endometrioid and non-endometrioid histologic subtypes [2]. Endometrioid is the predominant subtype, comprising 80 % of cases. However, the non-endometrioid subtypes, though less common, are more aggressive [3]. Most estimates of endometrial cancer incidence do not take into account hysterectomy prevalence. Not accounting for hysterectomy prevalence can inflate the denominator in incidence calculations, resulting in underestimates of the true incidence, given that women who have had a hysterectomy are no longer at risk for developing endometrial cancer [2,4–6]. Previous work has found that hysterectomy-corrected non-endometrioid subtypes rates were highest in non-Hispanic Blacks, followed by non-Hispanic Whites, Hispanics, and non-Hispanic Asian/Pacific Islanders, whereas endometrioid subtype rates were highest in non-Hispanic Whites, with similar rates observed in other racial/ethnic groups [2]. However, to our knowledge, there are no hysterectomy-corrected endometrial cancer incidence rates available for American Indians [7–9].

Hysterectomy prevalence varies by histological subtype, race/ethnicity and age, and has been changing over time within some subgroups [10–14]. A recent study showed that nationwide hysterectomy prevalence decreased among women aged ≥40 years from 2006 to 2016, particularly among non-Hispanic Black and Hispanic women aged 50 to 74 years [10]. For American Indian women, hysterectomy prevalence estimates have been relatively high in recent years, showing the highest prevalence among women aged ≥60 years compared to Hispanics, non-Hispanic Asians, non-Hispanic Blacks, and non-Hispanic Whites [14]. An observational study also suggested that American Indian women have a higher prevalence of hysterectomy compared to non-Hispanic Whites [15]. However, an earlier study using data from 1989 to 1992 indicated that American Indian women have either a similar or a lower hysterectomy proportion than non-Hispanic Whites, a higher hysterectomy proportion than Hispanics, and a lower hysterectomy proportion than African American women in certain age groups [13].

Because of sizable differences in both hysterectomy prevalence and incidence of endometrial cancers across histologic subtype, race/ethnicity, age, and time, the failure to account for hysterectomy status may lead to biased estimations of the disparities in endometrial cancer incidence rates among racial/ethnic groups. There is a notable gap in knowledge regarding hysterectomy-corrected endometrial cancer incidence, particularly among racial/ethnic minority groups like American Indians, and trends within age groups in recent years. Given that California is one of the states with the largest American Indian populations [16], a community that faces substantial health disparities [17,18], it is important to understand endometrial cancer incidence trends in this population.

To address this knowledge gap, we used data from the California Cancer Registry (CCR) and hysterectomy prevalence estimates from the Behavioral Risk Factor Surveillance System (BRFSS), to examine hysterectomy-adjusted incidence trends in endometrioid and non-endometrioid subtypes of endometrial cancer by race/ethnicity, and age, from 2012 to 2019, to provide a more comprehensive and accurate representation of the current endometrial cancer burden in California.

## 2. Methods

### 2.1. Data sources

#### 2.1.1. California Cancer Registry database

We identified endometrial cancer cases diagnosed January 1, 2012 through December 31, 2019 from the CCR to correspond to data in the BRFSS. The CCR is a state-mandated population-based cancer surveillance system that collects incidence reports on more than 170,000 new cases of cancer diagnosed annually in California. It is comprised of three National Cancer Institute’s Surveillance, Epidemiology, and End Results program registries that collect data on tumor characteristics, treatment, and patient demographic information [19]. Cases were classified by histologic subtypes defined by the third edition of the International Classification of Diseases for Oncology histology codes (Supplementary Table 1) [2]. We excluded women <25 years because of small numbers (*N* = 39,303) for trend analysis when stratified by histology, race/ethnicity and/or age, as well as women with unknown race/ethnicity (*N* = 21,675) due to population denominators were not available for this group.

#### 2.1.2. Behavioral Risk Factor Surveillance System

The BRFSS, a collaborative effort between the Centers for Disease Control and Prevention and each state health department, is a United States national self-report telephone survey that provides prevalence data concerning behavioral risk factors associated with the nation’s most common health conditions [20]. The BRFSS currently uses a dual-frame sampling design, with both cell and landline random-digit dial components. Survey-weighted estimates of hysterectomy prevalence were calculated for women age ≥ 25 years. Because BRFSS only obtains information on hysterectomy in even-numbered years after 2000 and there was a change in statistical weighting method beginning with the 2011 dataset, hysterectomy prevalence was imputed for the odd-numbered years after 2012 by calculating a population-weighted average of the neighboring years, following the methods of prior research [2,21].

### 2.2. Statistical analysis

Annual age-adjusted incidence rates, uncorrected for hysterectomy prevalence were calculated using SEER*Stat software (version 8.4.2) for endometrial cancer overall and by histologic subtype, stratified by year of diagnosis (2012 to 2019), 10-year age groups (i.e., 25 to 34, 35 to 44, 45 to 54, 55 to 64, and ≥ 65), and race/ethnicity (Non-Hispanic White, Non-Hispanic Black, Hispanic, Asian/Pacific Islander, and American Indian). Population data, used in the denominators, were obtained from the National Center for Health Statistics [22]. Rates were age adjusted to the 2000 US standard population and expressed per 100,000 woman-years. In addition to annual incidence rates, we also summarized incidence rates at 4 year intervals (2012–2015, 2016–2019), consistent with previous work [6].

We estimated smoothed survey-weighted hysterectomy prevalence using logistic regression with coefficients for year, 10-year age group, and race/ethnicity, using data from BRFSS. Prevalence estimates were predicted from the model, adjusted for year of diagnosis, age group, and race/ethnicity. These estimates were used to correct the populations at risk of endometrial cancer by removing the proportion of women with a hysterectomy from the denominator. To account for women with endometrial cancer who received a hysterectomy for treatment, we added those cases (identified from California Cancer Registry) back into the corrected denominator. Hysterectomy-corrected rates were age standardized to the 2000 US population. We estimated incidence rate ratios and 95 % confidence intervals (CIs) to compare incidence rates between groups and calculated the percent change between uncorrected and corrected rates.

Trends in endometrial cancer age-adjusted incidence rates were estimated using the National Cancer Institute’s Joinpoint regression software (version 5.0.1), which calculates annual percent change (APC) and 95 % CIs and uses *t*-tests to determine whether APCs are statistically significantly different from zero [23]. The program selects the best-fitting log-linear regression model to identify years when APCs significantly changed, providing a minimum number of joinpoints necessary to fit the data [24,25]. If more than one APC joinpoint trend was estimated, separate trends are shown. Trends were plotted using a semilogarithmic scale using R software (version 4.3.0).

## 3. Results

The hysterectomy-corrected incidence rate of the endometrioid subtype among women aged >25 showed a slight increase from 2012–2015 to 2016–2019 across all age and racial/ethnic groups (Table 1). Notably, endometrioid subtype incidence for American Indian women increased from 60.8 in 2012–2015 to 65.1 per 100,000 in 2016–2019. This increase was followed by Hispanic women, Asian/Pacific Islander women, non–Hispanic Black women, and non–Hispanic White women. For non-endometrioid subtypes incidence, there was a slight increase from 27.7 in 2012–2015 to 30.8 per 100,000 in 2016–2019 among non-Hispanic Black women. Similarly, among American Indian women, there was a slight increase from 12.9 in 2012–2015 to 15.2 per 100,000 in 2016–2019. The uncorrected incidence rate is shown in Supplementary Table 2.

**Fig. 1.**
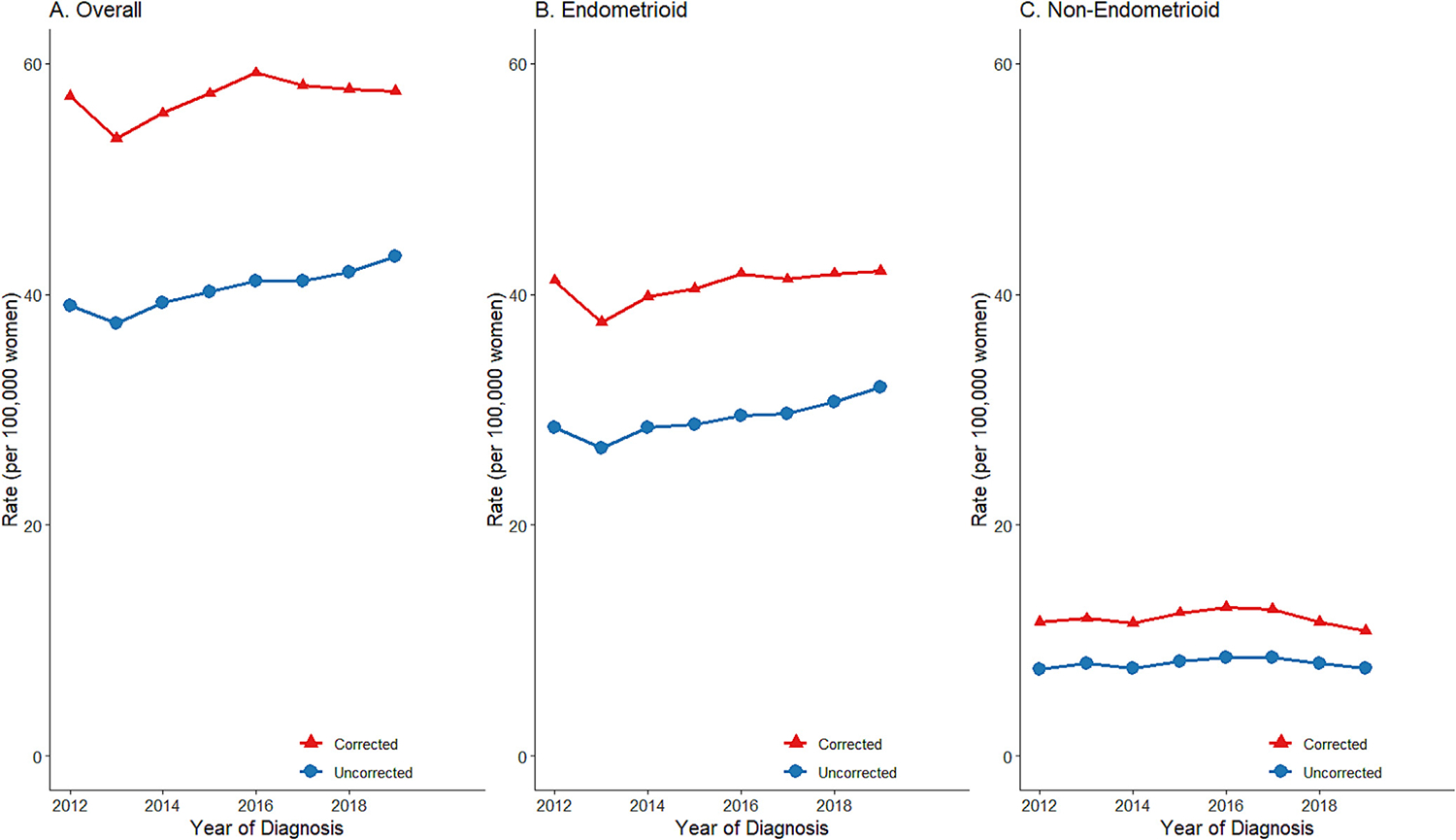
Trends in Hysterectomy–Uncorrected and -Corrected Age–Adjusted Incidence Rates of Endometrial Cancer Overall and by Histologic Subtype, California, 2012–2019.

**Fig. 2.**
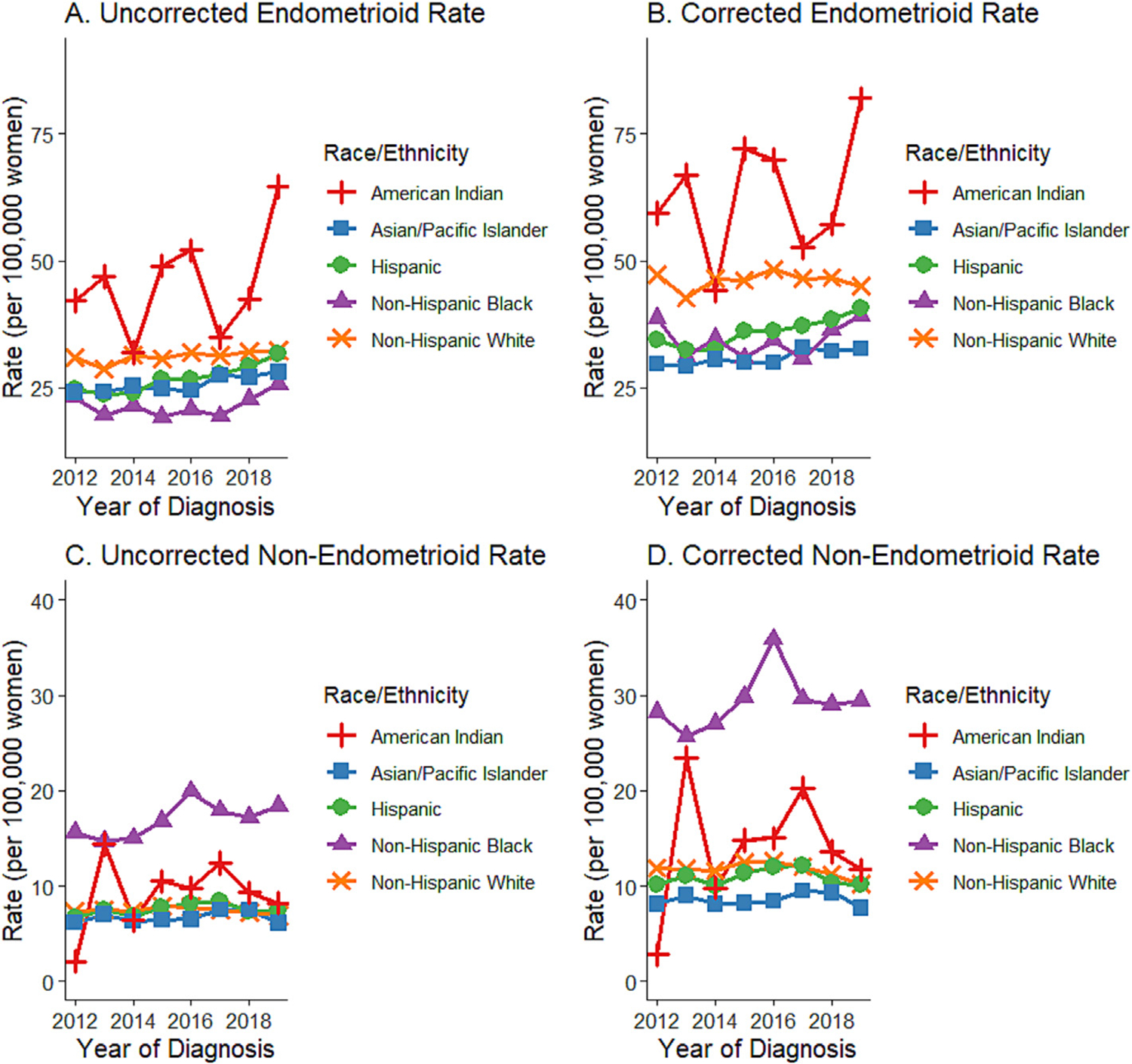
Trends in Race/Ethnicity Specific Incidence Rates of Endometrial Cancer by Histologic Subtype Uncorrected and Corrected for Hysterectomy Prevalence, California, 2012–2019.

From 2012 to 2019, the incidence rates corrected for hysterectomy consistently exceeded the uncorrected rates across all histologic subtypes and racial/ethnic groups within both endometrioid and non-endometrioid subtypes (Fig. 1 and 2). Notably, while the uncorrected endometrioid subtype incidence rate among non-Hispanic Black women appeared to be the lowest among all racial/ethnic groups, the corrected rates were found to be similar to those for Hispanic and Asian/Pacific Islander women.

The strength of association between race/ethnicity and incidence rate was influenced by hysterectomy prevalence (Table 2). The racial/ethnic differences in endometrioid subtype incidence between non-Hispanic White and Hispanic women, as well as between non-Hispanic White and Asian/Pacific Islander women, became larger after correction across all calendar periods. In contrast, the differences between non-Hispanic White women and non-Hispanic Black women, and between non-Hispanic White women and American Indian women, saw a slight reduction in magnitude throughout all calendar periods. For non-endometrioid subtype incidence corrected for hysterectomy prevalence, the racial/ethnic disparities widened between non-Hispanic White women and both non-Hispanic Black women and Asian/Pacific Islander women in all calendar periods. The difference in incidence rates between non-Hispanic White and Hispanic women increased slightly during the 2012–2015 period, with a subsequent reversal in rate ratio observed during 2016–2019. However, the racial/ethnic disparity between non-Hispanic White women and American Indian women remained nearly the same after correction. The hysterectomy prevalence trend by race/ethnicity is shown in Supplementary Fig. 1.

Endometrioid subtype incidence was highest in women aged 55–64 years prior to hysterectomy correction (Supplementary Fig. 2A). After correction, however, the highest incidence was observed in women ≥65 years (Supplementary Fig. 2B). An upward trend in corrected endometrioid subtype incidence was observed among women between ages 25 and 54 years from 2012 to 2019, though the age disparities diminished compared with uncorrected incidence (Table 3). For the non-endometrioid subtypes (Table 3 and Supplementary Fig. 2C, 2D), no significant trend was observed in corrected incidence among all age groups with the exception of women ≥65 years where the incidence increased starting from 2012, peaked in 2017, and then rapidly declined until 2019. The uncorrected annual percent change (APC) is shown in Supplementary Table 3.

Further, we looked at incidence trends within age groups among racial/ethnic categories (Table 4). Hysterectomy-corrected endometrioid subtype incidence increased in all age groups among Hispanics, with the exception of women aged ≥65 years. Notably, there was a dramatic surge (APC, 18.42 %; 95 % CI, 7.67 % to 38.49 %) observed in the youngest age group (25–34) among Hispanics from 2016 to 2019. Additionally, an upward trend (APC, 8.12 %, 95 % CI, 1.64 % to 18.63 %) was seen in non-Hispanic Black women aged between 55 and 64 years. In addition, Asian/Pacific Islander women aged ≥35 years seemed to experience an increasing trend in endometrioid subtype incidence from 2012 to 2019, though statistical significance was not reached. For non-endometrioid subtypes incidence, we observed a decreasing trend among non-Hispanic Whites (2017–2019: APC, −10.05 %; 95 % CI, −12.48 % to −6.42 %) and Blacks (2016–2019: APC, −8.16 %; 95 % CI, −23.24 % to −0.06 %) aged ≥65 years. Meanwhile, Hispanic women aged 25–34 also experienced a marked decline (2015–2019: APC, −31.04 %; 95 % CI, −59.59 % to −24.74 %), as did those aged 45–54 (2016–2019: APC, −16.68 %; −31.82 % to −8.08 %) and 55–64 (2017–2019: APC, −14.68 %; 95 % CI, −21.14 % to −6.22 %) years.

## 4. Discussion

Using hysterectomy-corrected incidence rates, our population-based study identified racial/ethnic disparities across age groups, including a recent decline in non-endometrioid subtypes incidence among certain populations. Specifically, we observed a decrease in non-endometrioid subtypes incidence among non-Hispanic Whites (2017–2019) and among non-Hispanic Blacks aged ≥65 years (2016–2019), as well as among Hispanic women in the 25–34 age group (2015–2019), 45–54 age group (2016–2019), and 55–64 age group (2017–2019). In addition, our study revealed that endometrioid subtype incidence is highest among American Indians, and increasing among younger populations, primarily driven by Hispanic patients. We also confirmed that uncorrected endometrial cancer incidence underestimates and distorts incidence trends across histologic subtype, racial/ethnic and age groups. Consistent with previous research [11,15], we found that endometrioid subtype incidence increased with age after adjusting for hysterectomy. However, before hysterectomy correction [11,15], we found women aged 55–64 had a higher incidence than those aged ≥65, highlighting the importance of accounting for hysterectomy prevalence, as suggested in previous research [4–6,15]. Corrected for hysterectomy, to the findings from our study uncover the unbiased racial/ethnic and age differences in endometrial cancer incidence trends among the California population in recent years, offering research directions for factors contributing to the incidence trends.

Previous studies in the US have examined racial/ethnic disparities in hysterectomy-corrected endometrial cancer incidence among various groups [2,11,15,26–29]. However, to the best of our knowledge, this study represents the first epidemiologic study investigating the hysterectomy-corrected incidence of endometrial cancer by histologic subtype in American Indians. Our findings of increasing endometrioid subtype incidence, particularly among Hispanic and Asian/Pacific Islander women, and no significant trend in non-Hispanic Whites, non-Hispanic Blacks and American Indians are partially consistent with prior studies [2,30]. One study found that endometrioid subtype incidence rose among non-Hispanic Blacks, Hispanics, and Asians/Pacific Islanders, but were stable among non-Hispanic Whites during 2000–2015 [2]. Another study found a decreasing trend in non-Hispanic White women, but found no trend in non-Hispanic Black women using data from 2000 to 2011 [30]. However, these studies used earlier years of data and did not correct for hysterectomy prevalence, which could explain the discrepancies.

Further, we found American Indians had the highest incidence of the endometrioid subtype. It has long been considered that endometrioid subtype rates were highest in non-Hispanic Whites [2,11,28], partly due to the sparse data on American Indians in the US. The elevated risk among American Indians compared to other racial/ethnic groups in California may stem from a complex interplay of obesity, physical inactivity, hypertension and diabetes [31–34]. According to BRFSS survey data, American Indian women have a higher prevalence of obesity (35.4 % vs. 21.5 %) and are less physically active (30.6 % vs 22 %) than non-Hispanic White women regardless of geographic region [35]. Another study also reported that American Indian, Hispanic and Black women were more likely to be overweight or obese than non-Hispanic Whites [36]. In addition, American Indian women have a higher prevalence of diabetes, a risk factor for endometrial cancer, than non-Hispanic White women (13.9 % vs 6.9 %) [35]. The increasing incidence of the endometrioid subtype among younger populations, particularly among Hispanic women, is concerning, as it indicates a rise in this cancer within the younger reproductive age group of Hispanic women. Additionally, in the US, Hispanic children show disproportionately high rates of obesity compared to children of other racial/ethnic groups [37,38].

In contrast to an increasing trend of the endometrioid subtype, our study found a sharp decline in non-endometrioid subtypes incidence among all women from 2017 to 2019, with differences by race/ethnicity and age. Particularly, non-endometrioid subtypes incidence is significantly decreasing among Hispanic. Differences in modifiable risk factors are important to consider when evaluating racial/ethnic disparities in the observed incidence patterns, where endometrioid subtype incidence is increasing while non-endometrioid subtypes are not. Oral contraceptive use, cigarette smoking, and reproductive factors are associated with endometrioid and non-endometrioid subtypes to similar extents [17,31,34,39,40]. However, the effect of obesity differs between the two subtypes [17,39,40]. Obesity has a stronger effect on the endometrioid than non-endometrioid subtypes [17,39], so the increasing obesity prevalence among Hispanics may not be impacting trends in the non-endometrioid subtypes [37]. Another consideration is the growing prevalence of risk-reducing hysterectomies [41] in women with *BRCA1* gene mutations, which was recently found to be associated with the non-endometrioid subytpes [42]. Although this likely impacts a small portion of the total population, it remains an important consideration.

We found that non-Hispanic Black women had the highest rates of non-endometrioid subtypes, which is consistent with prior research [2,43,44]. Notably, American Indians ranked second. Genomic studies have elucidated the occurrence of *p53* and *PIK3R1* mutations in Black women, which are likely related to the aggressive non-endometrioid subtypes and unfavorable prognosis [45,46]. However, to the best of our knowledge, no research has investigated risk factors for the non-endometrioid subtypes among American Indian women, who have been underrepresented in clinical trials of gynecologic cancer [47]. This may lead to worse outcomes for minority groups, such as American Indian patients, who are treated using the same paradigms based on clinical trials that included people largely socially and genetically different. Therefore, future studies are warranted to understand the factors contributing to the high incidence of the non-endometrioid subtypes incidence among American Indian women.

This study has some limitations. First, although BRFSS is the only nationally representative database with state-level hysterectomy prevalence estimates, response rates have been lower compared with other surveys. However, prior studies have found that BRFSS prevalence estimates are comparable to other national surveys that rely on self-reports [48,49]. Second, because the BRFSS collected hysterectomy information every other year, we had to estimate the prevalence for odd years by averaging the data from the neighboring years, which has been utilized by prior research [2,11,21,28]. Third, race/ethnicity are reported at an aggregate level in the population denominators, precluding more refined identification of racial/ethnic subgroups, including separating Asians from Pacific Islanders. Despite these limitations, our corrected incidence estimates enable unbiased comparisons over time and across diverse racial/ethnic groups, including American Indians.

To our knowledge, this study represents the first analysis of hysterectomy-corrected endometrial cancer incidence by histologic subtype and age, within California’s diverse population, and including American Indians. We found racial/ethnic disparities in endometrial cancer incidence, where non-Hispanic Black women show the highest rates of the non-endometrioid subtypes, followed by American Indians, who also exhibited the highest incidence of the endometrioid subtype. This finding, overlooked in previous research, underscores the need for future studies to elucidate the factors contributing to the notable pattern in non-endometrioid subtypes incidence among American Indians. Our study also reveals that endometrioid subtype incidence is increasing among younger Hispanic women. Disparities in the incidence of endometrial cancer persists despite advances in prevention strategies and suggest that more research is needed to better understand possible modifiable risk factors racial/ethnic minorities face to lower the risk of developing the disease.

## Supplementary Material

1

## Figures and Tables

**Table 1:** Endometrial Cancer Incidence Rates^#^ Corrected for Prior Hysterectomy by Histologic Subtype, California, 2012–2019.

Characteristic	N	Overall: 2012–2019	2012–2015	2016–2019
**Endometrioid**				
**Race/ethnicity**				
Non-Hispanic White	416,939	46.1 (45.5,46.8)	45.6 (44.7,46.6)	46.6 (45.6,47.5)
Non-Hispanic Black	40,405	34.5 (32.8, 36.3)	33.8 (31.3, 36.4)	35.2 (32.8, 37.7)
Hispanic	108, 664	36.2 (35.4, 37.0)	33.9 (32.7, 35.1)	38.2 (37.1, 39.3)
Asian/Pacific Islander	76, 687	31.0 (30.1, 31.9)	29.9 (28.6, 31.2)	32.0 (30.8, 33.2)
American Indian	3133	62.9 (55.7, 70.8)	60.8 (50.6, 72.5)	65.1 (55.1, 76.4)
**Age**				
25–34	16, 778	3.3 (3.1, 3.5)	3 (2.7, 3.4)	3.5 (3.2, 3.9)
35–44	40, 386	11.7 (11.2, 12.2)	10.9 (10.3, 11.6)	12.5 (11.8, 13.2)
45–54	85, 129	34.4 (33.5, 35.3)	33.5 (32.3, 34.7)	35.4 (34.1, 36.6)
55–64	134, 870	87.7 (86.1, 89.2)	86.3 (84.1, 88.6)	88.9 (86.8, 91.1)
≥65	372, 266	92.8 (91.3, 94.4)	91 (88.7, 93.4)	94.4 (92.2, 96.5)
**Non-endometrioid**				
**Race/ethnicity**				
Non-Hispanic White	57, 604	11.7 (11.4, 12.0)	12.0 (11.5, 12.5)	11.4 (11.0, 11.9)
Non-Hispanic Black	6324	29.4 (27.7, 31.1)	27.7 (25.3, 30.2)	30.8 (28.5, 33.2)
Hispanic	19, 893	10.9 (10.4, 11.4)	10.7 (9.9, 11.4)	11.2 (10.5, 11.8)
Asian/Pacific Islander	10, 112	8.5 (8.1, 9.0)	8.3 (7.7, 9.0)	8.7 (8.1, 9.4)
American Indian	580	14.1 (10.8, 18.1)	12.9 (8.4, 18.8)	15.2 (10.6, 21.1)
**Age**				
25–34	1364	0.2 (0.2, 0.3)	0.3 (0.2, 0.4)	0.2 (0.1, 0.2)
35–44	5236	1.0 (0.8, 1.1)	1.0 (0.8, 1.2)	1.0 (0.8, 1.2)
45–54	14, 590	4.3 (4.0, 4.6)	4.3 (3.9, 4.7)	4.3 (3.9, 4.8)
55–64	25, 697	21.1 (20.4, 21.9)	21.2 (20.1, 22.4)	21.0 (20.0, 22.1)
≥65	47, 934	40.3 (39.3, 41.4)	39.9 (38.4, 41.5)	40.6 (39.2, 42.1)

# Rates are per 100, 000 and age adjusted to the 2000 US standard population.

**Table 2:** Race/Ethnicity-Specific Rate Ratios (RR) of Hysterectomy-Uncorrected and -Corrected Incidence Rates^#^ of Endometrial Cancer by Histologic Subtype, California, 2012–2019.

	Uncorrected RR (95 % CI)		Corrected RR (95 % CI)	
	2012–2015	2016–2019	2012–2015	2016–2019
**Endometrioid**				
Non-Hispanic White	Ref	Ref	Ref	Ref
Non-Hispanic Black	0.68 (0.63, 0.74) *	0.70 (0.65, 0.75)*	0.74 (0.68, 0.80)*	0.76 (0.70, 0.81)*
Hispanic	0.82 (0.78, 0.85)*	0.91 (0.88, 0.94)*	0.74 (0.71, 0.77)*	0.82 (0.79, 0.85)*
Asian/Pacific Islander	0.81 (0.77, 0.85)*	0.84 (0.81, 0.88)*	0.65 (0.62, 0.69)*	0.69 (0.66, 0.72)*
American Indian	1.40 (1.16, 1.68)*	1.52 (1.28, 1.79)*	1.33 (1.11, 1.59)*	1.40 (1.18, 1.64)*
**Non-endometrioid**				
Non-Hispanic White	Ref	Ref	Ref	Ref
Non-Hispanic Black	2.07 (1.88, 2.28)*	2.50 (2.29, 2.72)*	2.31 (2.10, 2.54)*	2.70 (2.47, 2.94)*
Hispanic	0.96 (0.88, 1.03)	1.06 (0.99, 1.14)	0.89 (0.82, 0.96)*	0.98 (0.91, 1.05)
Asian/Pacific Islander	0.86 (0.79, 0.95)*	0.94 (0.87, 1.03)	0.70 (0.63, 0.76)*	0.76 (0.70, 0.83)*
American Indian	1.12 (0.73, 1.65)	1.35 (0.94, 1.90)	1.07 (0.70, 1.58)	1.33 (0.92, 1.85)

RR: rate ratios.

# Rates are per 100, 000 and age adjusted to the 2000 US standard population

* *P* < 0.05.

**Table 3:** Time Period and Corresponding Annual Percent Changes (APC) in Hysterectomy–Uncorrected and -Corrected Endometrial Cancer Incidence by Histologic Subtype, California, 2012–2019.

	Uncorrected Trend 1		Uncorrected Trend 2		Corrected Trend 1		Corrected Trend 2	
	Time Period	APC (95 %CI)	Time Period	APC (95 %CI)	Time Period	APC (95 %CI)	Time Period	APC (95 %CI)
**Histologic subtype**								
Endometrioid	2012–2019	2.11 (1.22, 3.03)*			2012–2019	1.01 (0.04, 2.04)*		
Non-endometrioid	2012–2017	2.49 (1.45, 4.52)*	2017–2019	−5.92 (−9.17, −1.88)*	2012–2017	2.18 (1.13, 3.93)*	2017–2019	−8.24 (−11.06, −4.69)*
**Endometrioid**								
**Race/ethnicity**								
Non-Hispanic White	2012–2019	1.12 (−0.04, 2.29)			2012–2019	0.15 (−1.07, 1.41)		
Non-Hispanic Black	2012–2017	−2.18 (−10.74, 1.18)	2017–2019	15.09 (3.48, 24.29)*	2012–2017	−2.66 (−14.33, 6.31)	2017–2019	12.47 (−0.88, 24.94)
Hispanic	2012–2019	4.09 (2.35, 6.11)*			2012–2019	3.02 (1.41, 4.98)*		
Asian/Pacific Islander	2012–2019	2.33 (1.40, 3.38)*			2012–2019	1.69 (0.48, 3.05)*		
American Indian	2012–2019	4.14 (−2.53, 12.15)			2012–2019	2.79 (−4.15, 11.17)		
**Age, years**								
25–34	2012–2016	−0.05 (−7.43, 3.82)	2016–2019	13.71 (7.55, 24.55)*	2012–2016	−1.08 (−9.76, 3.40)	2016–2019	14.10 (6.93, 26.58)*
35–44	2012–2017	1.23 (−6.72, 5.23)	2017–2019	13.46 (3.76, 22.26)*	2012–2019	3.73 (0.13, 7.69)*		
45–54	2012–2019	2.51 (0.61, 4.55)*			2012–2019	1.70 (0.32, 3.12)*		
55–64	2012–2019	1.40 (0.82, 2.02)*			2012–2019	0.55 (−0.42, 1.58)		
≥65	2012–2019	1.52 (0.16, 2.99)*			2012–2019	0.29 (−1.40, 2.14)		
**Non-endometrioid**								
**Race/ethnicity**								
Non-Hispanic	2012–2016	1.86 (0.55, 4.18)*	2016–2019	−4.20	2012–2016	2.05 (0.26, 4.56)*	2016–2019	−6.47
White				−4.20 (−7.33, −2.22)*				−6.47 (−9.99, −4.08)*
Non-Hispanic	2012–2019	3.14 (−0.23, 6.99)			2012–2019	1.46 (−1.62, 5.52)		
Black								
Hispanic	2012–2017	3.86 (1.83, 11.14)*	2017–2019	−6.57 (−12.16, −0.19)*	2012–2017	3.31 (1.27, 9.01)*	2017–2019	−9.14 (−14.55, −2.15)*
Asian/Pacific Islander	2012–2019	1.38 (−2.79, 6.25)			2012–2019	0.34 (−3.67, 4.85)		
American Indian	2012–2019	−2.30 (−15.81, 15.47)			2012–2019	−3.18 (−19.17, 18.89)		
**Age, years**								
25–34	2012–2019	−8.48 (−16.91, −2.13)*			2012–2019	NA		
35–44	2012–2019	0.28 (−15.79, 18.04)			2012–2019	−2.13 (−15.80, 13.75)		
45–54	2012–2019	0.52 (−5.81, 7.19)			2012–2019	−0.42 (−7.05, 7.29)		
55–64	2012–2019	−0.45 (−2.64, 1.79)			2012–2017	0.86 (−3.40, 10.42)	2017–2019	−7.98 (−15.67, 0.24)
≥65	2012–2016	4.02 (2.03, 11.70)*	2016–2019	−2.63 (−8.93, 0.16)	2012–2017	2.48 (1.50, 4.32)*	2017–2019	−7.99 (−11.39, −3.84)*

Abbreviation: NA, not applicable.

The joinpoints are the years where statistically significant changes in incidence trends occur, identified by fitting a segmented regression model to the data; the APC is then calculated for each segment between joinpoints.

* Indicates that the Annual Percent Change (APC) is significantly different from zero at the alpha = 0.05 level.

**Table 4:** Time Period and Corresponding Annual Percent Change (APC) in Hysterectomy–Corrected Endometrial Cancer Incidence by Histologic Subtype, Race/Ethnicity and Age, California, 2012–2019.

	Endometrioid		Endometrioid		Non-Endometrioid		Non-Endometrioid	
	Trend 1		Trend 2		Trend 1		Trend 2	
	Time Period	APC (95 %CI)	Time Period	APC (95 %CI)	Time Period	APC (95 %CI)	Time Period	APC (95 %CI)
**Non-Hispanic White**								
25–34	2012–2019	3.01 (−4.25, 11.72)			2012–2019	NA		
35–44	2012–2019	−0.06 (−5.24, 5.51)			2012–2019	−11.29 (−31.38, 6.51)		
45–54	2012–2019	0.28 (−1.87, 2.41)			2012–2019	−2.78 (−9.97, 4.45)		
55–64	2012–2017	0.43 (−2.08, 5.45)	2017–2019	−3.31 (−7.71, 0.79)	2012–2019	−4.01 (−7.24, −0.77)*		
≥65	2012–2019	0.34 (−1.62, 2.53)			2012–2017	2.72 (1.68, 4.09)*	2017–2019	−10.05 (−12.48, −6.42)*
**Non-Hispanic Black**								
25–34	2012–2017	2.02 (−44.47, 257.62)	2017–2019	−64.22 (−95.67, 31.65)	2012–2019	NA		
35–44	2012–2019	−1.63 (−15.08, 14.22)			2012–2019	NA		
45–54	2012–2019	1.43 (−6.73, 10.52)			2012–2019	0.85 (−15.54, 21.09)		
55–64	2012–2016	−4.57 (−13.47, −0.06)*	2016–2019	8.12 (1.64, 18.63)*	2012–2019	1.43 (−3.74, 7.53)		
≥65	2012–2019	1.41 (−4.82, 9.02)			2012–2016	9.39 (3.08, 33.34)*	2016–2019	−8.16 (−23.24, −0.06)*
**Hispanic**								
25–34	2012–2016	−1.23 (−16.18, 7.11)	2016–2019	18.42 (7.67, 38.49)*	2012–2015	−2.05 (−15.51, 37.73)	2015–2019	−31.04 (−59.59, −24.74)*
35–44	2012–2019	6.35 (0.89, 12.72)*			2012–2019	2.08 (−8.93, 15.51)		
45–54	2012–2019	3.42 (0.66, 6.61)*			2012–2016	11.33 (4.12, 26.85) *	2016–2019	−16.68 (−31.82, −8.08)*
55–64	2012–2019	3.00 (0.47, 5.97)*			2012–2017	8.67 (6.15, 13.17)*	2017–2019	−14.68 (−21.14, −6.22)*
≥65	2012–2014	−1.94 (−5.52, 3.30)	2014–2019	2.57 (−0.90, 6.63)	2012–2019	−0.38 (−3.24, 2.90)		
**Asian/Pacific Islander**								
25–34	2012–2019	4.02 (−5.90, 15.93)			2012–2019	NA		
35–44	2012–2019	3.11 (−0.08, 6.84)			2012–2019	3.12 (−11.90, 22.86)		
45–54	2012–2019	1.54 (−0.71, 4.00)			2012–2019	1.72 (−6.92, 12.27)		
55–64	2012–2019	1.67 (−0.12, 3.65)			2012–2019	−1.59 (−5.10, 2.28)		
≥65	2012–2019	1.02 (−0.73, 3.11)			2012–2019	1.02 (−3.13, 5.95)		
**American Indian**								
25–34	2012–2019	NA			2012–2019	NA		
35–44	2012–2019	−0.74 (−25.13, 30.42)			2012–2019	NA		
45–54	2012–2019	7.14 (−7.73, 25.34)			2012–2019	NA		
55–64	2012–2019	7.39 (−5.59, 24.26)			2012–2019	NA		
≥65	2012–2019	−1.88 (−14.66, 14.03)			2012–2019	0.95 (−17.36, 29.09)		

Abbreviation: NA, not applicable.

The joinpoints are the years where statistically significant changes in incidence trends occur, identified by fitting a segmented regression model to the data; the APC is then calculated for each segment between joinpoints.

* Indicates that the Annual Percent Change (APC) is significantly different from zero at the alpha = 0.05 level.

## Data Availability

The data that support the findings of this study are available from the California Cancer Registry. Access is granted through an application process by the management or data custodians (https://www.ccrcal.org/retrieve-data/).

## HIGHLIGHTS

- Our study uncovers that endometrioid subtype incidence is increasing among younger Hispanic women.
- American Indians exhibited the highest incidence of the endometrioid subtype.
- Non-Hispanic Black women show the highest rates of the non-endometrioid subtypes, followed by American Indians.
- This study underscores the importance of jointly examining racial/ethnic disparities with age and histologic subtype.
- More research is needed to better understand possible modifiable risk factors racial/ethnic minorities face.

## Appendix A. Supplementary data

Supplementary data to this article can be found online at https://doi.org/10.1016/j.ygyno.2025.04.581.
