# Supplementary material for: Disparities in hysterectomy-corrected endometrial cancer incidence trends by histologic subtype among racial/ethnic groups in California, 2012–2019
Source: Gynecol Oncol. Author manuscript; Available in PMC 2026 May 20. (PMC13189184; doi:10.1016/j.ygyno.2025.04.581)

**Supplementary Table 1.** Definition of Endometrioid and Non-Endometrioid Subtype using International Classification of Diseases for Oncology, Third Edition (ICD-O-3) Histology Codes

| **Endometrioid** |
| --- |
| 8050: Papillary carcinoma, NOS |
| 8141: Scirrhous adenocarcinoma |
| 8210: Adenocarcinoma in adenomatous polyp |
| 8211: Tubular adenocarcinoma |
| 8260: Papillary adenocarcinoma, NOS |
| 8261: Adenocarcinoma in villous adenoma |
| 8262: Villous adenocarcinoma |
| 8263: Adenocarcinoma in tubulovillous adenoma |
| 8380: Endometrioid carcinoma |
| 8381: Endometrioid adenofibroma, malignant |
| 8382: Endometrioid adenocarcinoma, secretory variant |
| 8383: Endometrioid adenocarcinoma, ciliated cell variant |
| 8440: Cystadenocarcinoma, NOS |
| 8470: Mucinous cystadenocarcinoma, NOS |
| 8471: Papillary mucinous cystadenocarcinoma |
| 8480: Mucinous adenocarcinoma |
| 8481: Mucin-producing adenocarcinoma |
| 8490: Signet ring cell carcinoma |
| 8560: Adenosquamous carcinoma |
| 8570: Adenocarcinoma with squamous metaplasia |
| 8571: Adenocarcinoma with cartilaginous and osseous metaplasia |
| 8140: Adenocarcinoma, NOS |
| **Non-endometrioid** |
| 8255: Adenocarcinoma with mixed subtypes |
| 8310: Clear cell adenocarcinoma, NOS |
| 8323: Mixed cell adenocarcinoma |
| 8441: Serous cystadenocarcinoma, NOS |
| 8460: Papillary serous cystadenocarcinoma |
| 8461: Serous surface papillary carcinoma |
| 8950: Mullerian mixed tumor |
| 8951: Mesodermal mixed tumor |
| 8980: Carcinosarcoma, NOS |
| 8981: Carcinosarcoma, embryonal |

**Supplementary Table 2.** Endometrial Cancer Incidence Rates^#^, Uncorrected for Prior Hysterectomy by Histologic Subtype and Time Period, California, 2012–2019

| **Characteristic** | **Overall: 2012–2019** | **2012–2015** | **2016–2019** |
| --- | --- | --- | --- |
| **Endometrioid** |  |  |  |
| **Race/ethnicity** |  |  |  |
| Non–Hispanic White | 31.2 (30.7, 31.7) | 30.4 (29.8, 31.1) | 31.9 (31.2, 32.6) |
| Non–Hispanic Black | 21.5 (20.5, 22.7) | 20.8 (19.3, 22.4) | 22.2 (20.7, 23.8) |
| Hispanic | 27.0 (26.4, 27.6) | 24.8 (24.0, 25.7) | 28.9 (28.1, 29.8) |
| Asian/Pacific Islander | 25.8 (25.0, 26.5) | 24.6 (23.6, 25.6) | 26.8 (25.8, 27.9) |
| American Indian | 45.5 (40.2, 51.3) | 42.6 (35.4, 50.9) | 48.4 (40.7, 57.0) |
| **Age** |  |  |  |
| 25–34 | 3.4 (3.1, 3.6) | 3.1 (2.7, 3.4) | 3.7 (3.3, 4.0) |
| 35–44 | 11.1 (10.7, 11.6) | 10.3 (9.6, 10.9) | 12.0 (11.3, 12.7) |
| 45–54 | 28.6 (27.9, 29.3) | 27.4 (26.4, 28.4) | 29.8 (28.7, 30.8) |
| 55–64 | 64.4 (63.3, 65.6) | 62.6 (60.9, 64.2) | 66.1 (64.5, 67.8) |
| ≥65 | 57.1 (56.1, 58.1) | 54.8 (53.4, 56.2) | 59.2 (57.8, 60.6) |
| **Non–endometrioid** |  |  |  |
| **Race/ethnicity** |  |  |  |
| Non–Hispanic White | 7.4 (7.2, 7.7) | 7.5 (7.2, 7.8) | 7.3 (7.0, 7.6) |
| Non–Hispanic Black | 17.0 (16.1, 18.0) | 15.6 (14.3, 17.0) | 18.3 (17.0, 19.8) |
| Hispanic | 7.5 (7.2, 7.9) | 7.2 (6.7, 7.7) | 7.8 (7.4, 8.3) |
| Asian/Pacific Islander | 6.7 (6.4, 7.1) | 6.5 (6.0, 7.0) | 6.9 (6.4, 7.5) |
| American Indian | 9.2 (7.0, 11.9) | 8.4 (5.5, 12.4) | 9.9 (6.9, 13.9) |
| **Age** |  |  |  |
| 25–34 | 0.2 (0.2, 0.3) | 0.3 (0.2, 0.4) | 0.2 (0.1, 0.3) |
| 35–44 | 0.9 (0.8, 1.1) | 0.9 (0.7, 1.1) | 0.9 (0.7, 1.1) |
| 45–54 | 3.6 (3.3, 3.8) | 3.5 (3.1, 3.9) | 3.6 (3.3, 4.0) |
| 55–64 | 15.4 (14.8, 15.9) | 15.3 (14.5, 16.1) | 15.4 (14.7, 16.2) |
| ≥65 | 25.1 (24.5, 25.8) | 24.3 (23.4, 25.3) | 25.8 (24.9, 26.7) |

^#^Rates are per 100,000 and age adjusted to the 2000 US standard


**Supplementary Table 3.** Time Period and Corresponding Annual Percent Change (APC) in Hysterectomy–Uncorrected Endometrial Cancer Incidence by Histologic Subtype, Race/Ethnicity and Age, California, 2012–2019

|  | **Endometrioid Trend 1** | | **Endometrioid Trend 2** | | **Non–endometrioid Trend 1** | | **Non–Endometrioid Trend 2** | |
| --- | --- | --- | --- | --- | --- | --- | --- | --- |
|  | **Time Period** | **APC (95%CI)** | **Time Period** | **APC (95%CI)** | **Time Period** | **APC (95%CI)** | **Time Period** | **APC (95%CI)** |
| **Non–Hispanic White** |  |  |  |  |  |  |  |  |
| 25–34 | 2012–2019 | 2.59 (-4.37, 11.14) |  |  | 2012–2019 | NA |  |  |
| 35–44 | 2012–2019 | 0.73 (-4.57, 6.24) |  |  | 2012–2019 | -9.51 (-28.67, 9.28) |  |  |
| 45–54 | 2012–2019 | 1.16 (-1.37, 3.65) |  |  | 2012–2019 | -1.71 (-7.82, 4.22) |  |  |
| 55–64 | 2012–2019 | 0.41 (-0.70, 1.55) |  |  | 2012–2019 | -3.42 (-6.26, -0.70) ^*^ |  |  |
| ≥65+ | 2012–2019 | 1.49 (-0.58, 3.78) |  |  | 2012–2017 | 3.03 (1.64, 7.18) ^*^ | 2017–2019 | -6.50 (-10.90, -1.33) ^*^ |
| **Non–Hispanic Black** |  |  |  |  |  |  |  |  |
| 25–34 | 2012–2019 | -7.74 (-31.05, 18.66) |  |  | 2012–2019 | NA |  |  |
| 35–44 | 2012–2019 | -1.32 (-15.03, 14.70) |  |  | 2012–2019 | NA |  |  |
| 45–54 | 2012–2019 | 2.13 (-4.92, 9.97) |  |  | 2012–2019 | 1.12 (-15.61, 21.58) |  |  |
| 55–64 | 2012–2016 | -4.26 (-14.92, 1.33) | 2016–2019 | 10.20 (2.27, 23.12) ^*^ | 2012–2019 | 2.33 (-3.12, 8.63) |  |  |
| ≥65 | 2012–2017 | -2.93 (-22.15, 18.34) | 2017–2019 | 21.37 (-1.79, 44.99) | 2012–2016 | 10.27 (1.62, 37.72) ^*^ | 2016–2019 | -4.90 (-21.85, 5.02) |
| **Hispanic** |  |  |  |  |  |  |  |  |
| 25–34 | 2012–2016 | 0.47 (-10.37, 6.43) | 2016–2019 | 18.23 (9.84, 32.89) ^*^ | 2012–2015 | -3.40 (-17.04, 30.15) | 2015–2019 | -30.71 (-54.69, -23.48) ^*^ |
| 35–44 | 2012–2019 | 6.60 (1.28, 12.96) ^*^ |  |  | 2012–2019 | 1.33 (-9.02, 13.73) |  |  |
| 45–54 | 2012–2019 | 3.90 (0.74, 7.54) ^*^ |  |  | 2012–2016 | 11.72 (4.56, 28.69) ^*^ | 2016–2019 | -15.53 (-30.03, -7.07) ^*^ |
| 55–64 | 2012–2019 | 3.90 (1.27, 7.02) ^*^ |  |  | 2012–2017 | 9.10 (6.78, 13.03) ^*^ | 2017–2019 | -13.10 (-18.82, -5.37) ^*^ |
| ≥65 | 2012–2015 | 0.39 (-3.64, 3.43) | 2015–2019 | 4.35 (2.39, 8.16) ^*^ | 2012–2019 | 0.79 (-1.38, 3.27) |  |  |
| **Asian/Pacific Islander** |  |  |  |  |  |  |  |  |
| 25–34 | 2012–2019 | 3.94 (-6.76, 16.65) |  |  | 2012–2019 | NA |  |  |
| 35–44 | 2012–2019 | 3.46 (-0.05, 7.40) |  |  | 2012–2019 | 2.07 (-11.87, 19.33) |  |  |
| 45–54 | 2012–2019 | 2.08 (0.04, 4.30) ^*^ |  |  | 2012–2019 | 2.19 (-6.17, 12.58) |  |  |
| 55–64 | 2012–2019 | 2.29 (0.47, 4.32) ^*^ |  |  | 2012–2019 | -1.39 (-5.04, 2.58) |  |  |
| ≥65 | 2012–2019 | 1.79 (-1.42, 5.49) |  |  | 2012–2019 | 2.07 (-2.14, 7.11) |  |  |
| **American Indian** |  |  |  |  |  |  |  |  |
| 25–34 | 2012–2019 | NA |  |  | 2012–2019 | NA |  |  |
| 35–44 | 2012–2019 | -0.67 (-25.25, 30.80) |  |  | 2012–2019 | NA |  |  |
| 45–54 | 2012–2019 | 7.83 (-4.20, 22.27) |  |  | 2012–2019 | NA |  |  |
| 55–64 | 2012–2019 | 8.38 (-4.11, 24.64) |  |  | 2012–2019 | NA |  |  |
| ≥65 | 2012–2019 | 0.40 (-11.70, 15.39) |  |  | 2012–2019 | 2.91 (-15.43, 31.03) |  |  |

Abbreviation: NA, not applicable

^*^ Indicates that the Annual Percent Change (APC) is significantly different from zero at the alpha = 0.05 level.

The joinpoints are the years where statistically significant changes in incidence trends occur, identified by fitting a segmented regression model to the data; the APC is then calculated for each segment between joinpoints.

**Supplementary Fig. 1.** Hysterectomy Prevalence Trends among California Women, 2012–2019


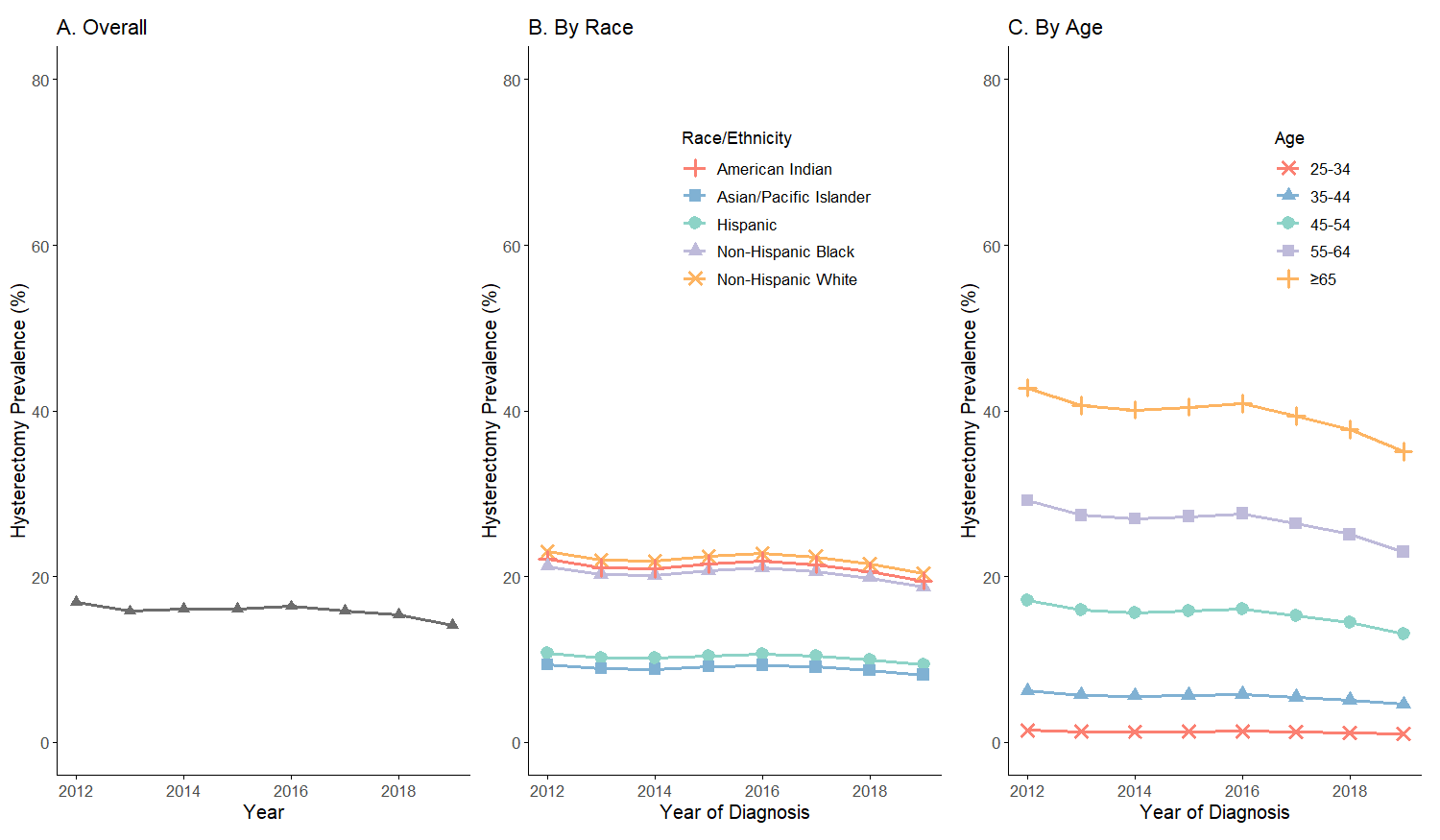


**Supplementary Fig. 2.** Trends in Age– Specific Incidence Rates of Endometrial Cancer by Histologic Subtype Uncorrected and Corrected for Hysterectomy Prevalence, California, 2012–2019


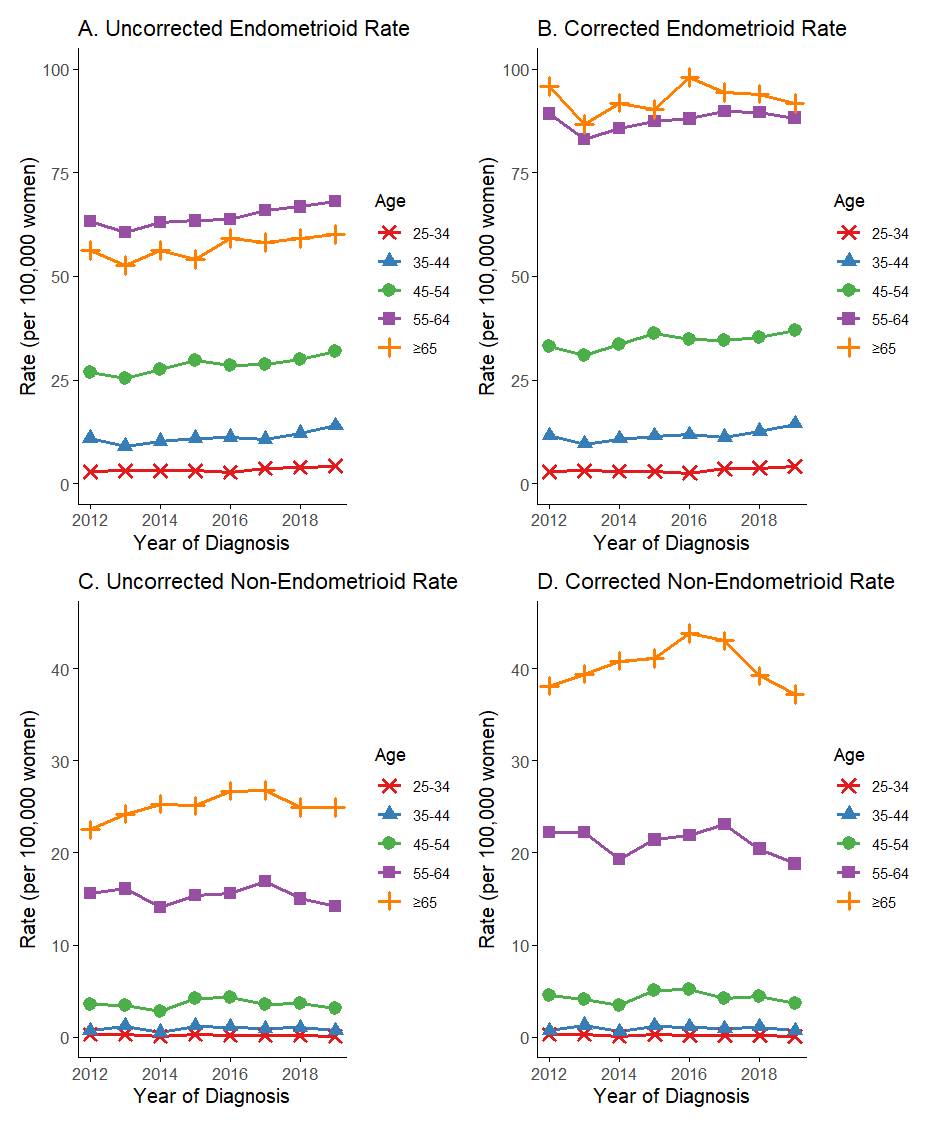

Supplement: 1 [file NIHMS2174389-supplement-1.docx]
